# Clinical characteristics, treatment and prognosis of children with SARS-CoV-2 infection complicated with severe neurological dysfunctions in Foshan, China

**DOI:** 10.3389/fped.2024.1414023

**Published:** 2024-08-16

**Authors:** Xiaoqian Chen, Yongqi Liang, Suhua Jiang

**Affiliations:** Department of Pediatrics, First People’s Hospital of Foshan, Guangdong, China

**Keywords:** SARS-CoV-2, pediatric patients, severe neurological impairment, neurological damage, warning indicators

## Abstract

**Objective:**

To analyze the clinical characteristics, treatment, and prognosis of children infected with SARS-CoV-2 following the adjustment of COVID-19 prevention and control policies in China in December 2022.

**Methods:**

A retrospective study was conducted on 9 cases of severe neurological dysfunction caused by SARS-CoV-2 infection in children admitted to Foshan First People's Hospital from December 17 to 31, 2022.

**Results:**

Among the 9 cases, 7 (71.43%) were under 3 years old, and 2 (22.2%) were over 3 years old with underlying diseases. All patients presented with fever and neurological symptoms such as consciousness disturbance and/or convulsions, and their conditions deteriorated rapidly within 24 h after the onset of fever, without respiratory symptoms. Levels of IL-6, LDH, and d-dimer were significantly elevated. Five cases died within 48 h of admission, one case died after 7 days of treatment due to secondary bacterial infection, and three cases survived for more than 48 h after the initial rescue. All patients developed rapid shock, and five cases experienced multi-organ failure within a short period. In terms of treatment, glucocorticoids were used in 5 cases, intravenous immunoglobulin (IVIG) in 3 cases, and blood purification and tocilizumab in 2 cases.

**Conclusion:**

SARS-CoV-2 infection in children can lead to severe neurological damage. High fever, convulsions, and inflammatory factors serve as early warning indicators. Glucocorticoids, immunoglobulins, blood purification, and tocilizumab may have some therapeutic effects, but further research is needed to confirm the efficacy.

## 1 Introduction

Since the emergence of the *coronavirus* disease 2019 (COVID-19) pandemic caused by severe acute respiratory disease *coronavirus* 2 (SARS-CoV-2) in China, numerous studies have focused on severe cases. However, there is a paucity of case reports or series that document neurological complications, such as seizures and encephalopathy, especially in pediatric patients. Despite the fact that children infected with SARS-CoV-2 typically exhibit mild or no symptoms, serious neurological involvement has been reported (1). Previous articles suggest that children infected with the Alpha or Delta strains mainly exhibit symptoms such as fever, cough, expectoration, and fatigue (2, 3), while those infected with the Omicron strain were significantly associated with neurological symptoms, especially the rapid onset of acute fulminant cerebral edema. Additionally, some children infected with the Omicron strain have been diagnosed with acute necrotizing encephalopathy (ANEC), which leads to a rapid death or causes serious neurological sequelae (4). ANEC is a disease characterized by respiratory or gastrointestinal infections, accompanied by high fever, rapid changes in consciousness and seizures. The mortality rate can be as high as more than 30%, and the proportion of complete recovery is about 10%.

The manifestations of the nervous system involvement of the novel *coronavirus* are diverse and can be divided into two categories: (1) non-specific manifestations such as headache, olfactory or taste loss, myalgia, fatigue, irritability, drowsiness and so forth. (2) Specific performance according to the different parts of the involvement is divided into peripheral nerve involvement, central nervous system involvement. The nervous system involvement of children with novel *coronavirus* infection is mainly non-specific, and the prognosis is good (5). A meta-analysis of 26 studies (including 3,707 children with novel *coronavirus* infection) by Panda et al. showed that 16.7% of children had non-specific neurological symptoms (headache, myalgia, fatigue, etc.), while the incidence of specific neurological manifestations was much lower. About 1% of children had manifestations such as encephalopathy and seizures (5). A report of 1,334 children with novel *coronavirus* infection in the UK found that there were 51 cases (3.8%) of children with specific neurological manifestations (encephalopathy, encephalitis, seizures, Guillain-Barre syndrome, acute disseminated encephalomyelitis, etc.), of which 25 cases were in line with MIS-C. In the non-MIS-C group, most of them showed single neurological symptoms or diseases such as status convulsion, Guillain-Barre syndrome, acute disseminated encephalomyelitis and so forth (6). In general, the neurological manifestations of COVID-19 in children are mainly specific neurological diseases such as status epilepticus, encephalopathy, meningeal signs, which are basically consistent with the content of this study.

This study aims to summarize the clinical and laboratory features, as well as the treatment strategies for severe neurological impairment among children infected with SARS-CoV-2 since the change in epidemic prevention policy in China in December 2022. Therefore, it is expected to provide a reference for clinical treatment of similar patients in the future.

## 2 Methods

The institutional review board of the First People's Hospital of Foshan approved this retrospective study. A total of 9 pediatric patients admitted with severe neurological impairment after SARS-CoV-2 infection from December 17, 2022 to December 31, 2022 were recruited. Eligible patients were unvaccinated and tested positive for COVID-19 antigen rapid test and/or COVID-19 nucleic acid test at the emergency department. Acute fulminant cerebral edema was defined as encephalopathy, followed by rapid progression to diffuse cerebral edema with herniation or herniation with brainstem compression on neuroimaging, with no other recognized causes of cerebral edema (e.g., organic, metabolic, toxic) (7, 8). Data collection included demographics, clinical symptoms, laboratory indicators, neuroimaging results, primary treatments, and outcomes. Descriptive analysis was conducted for clinical characteristics, and results were presented as counts (proportion).

Inclusion criteria: children aged 29 days to 15 years old, who were tested positive for novel coronavirus nucleic acid by nasopharyngeal swab rapid nucleic acid test, had neurological symptoms, and the disease progressed to disturbance of consciousness within 24 h. Exclusion criteria: previous underlying diseases, including but not limited to cerebral palsy, malnutrition, congenital immunodeficiency disease, etc. The clinical features were expressed as descriptive statistics, and the data were expressed as percentage (%).

The treatment plan included glucocorticoids, which were treated with shock therapy, with a dose of 30 mg/kg.d within 3 days, and then gradually reduced according to the condition; iVIG, according to the dose of 2 g/kg for shock therapy; blood purification (plasma exchange once, continuous CRRT after plasma exchange of 60 ml/kg); tocilizumab was administered at a dose of 12 mg/kg.

## 3 Results

Out of the nine cases, seven (71.43%) were under 3 years of age, ranging from 1 year and 2 months to 2 years and 9 months. The remaining two cases were older than 3 years and had underlying diseases, namely, nephrotic syndrome (6 years old) and cerebral palsy (4 years and 6 months old). All patients presented with fever and neurological symptoms such as disorders of consciousness and/or generalized tonic-clonic (GTC) seizures. Additionally, all children rapidly developed fulminant cerebral edema within 24 h of fever onset, without any respiratory symptoms such as cough. While three patients survived more than 48 h after the first rescue treatment, five died within 48 h of hospitalization, and one died due to secondary bacterial infection after 7 days of treatment. All patients suffered from shock rapidly after onset, and 5 patients developed multiple organ failure (respiratory, heart, liver, kidney and skeletal muscle) and disseminated intravascular coagulation (DIC) following a very short time.

Table 1 summarizes the manifestations of SARS-CoV-2 infection in the nine children and their main treatment regimen. In terms of in-hospital treatments, all cases received assisted mechanical ventilation. Glucocorticoids (i.e., methylprednisolone) were administered in five cases and three cases received intravenous immunoglobulin (IVIG) at a dose of 2 g/kg. A lumbar puncture was performed in one patient, and the cerebrospinal fluid (CSF) analysis revealed normal CSF white blood cell count but increased CSF protein level (1 g/L). Polymerase chain reaction (PCR) for SARS-CoV-2 in the CSF was negative. Cranial computed tomography (CT) was conducted in five patients, revealing significant cerebral edema. In all patients, the levels of inflammatory biomarkers, including interleukin-6 (IL-6), serum ferritin, lactate dehydrogenase (LDH), and D-dimer, significantly increased within the first three days of hospitalization.

**Table 1:** Manifestations summary of SARS-CoV-2 infection in the nine children and their main treatment regimen.

Variables		Case 1	Case 2	Case 3	Case 4	Case 5	Case 6	Case 7	Case 8	Case 9
Age		1 yr 2 mon	1 yr 2 mon	2 yr 9 mon	2 yr 2 mon	6 yr	1 yr 4 mon	4 yr 6 mon	1 yr 7 mon	2 yr
Gender		Male	Male	Female	Female	Male	Male	Female	Female	Male
Medical history						Nephrotic syndrome		Cerebral palsy		
Clinical evaluation										
	PICS	76	74	64	76	78	92	66	80	72
	PRISM III	23	40	39	39	18	14	38	12	5
Hospital stay		5 h	19 h	6 h	8 h	6 h	8 days	2.5 h	8 days	20 days
Clinical outcome		Death	Death	Death	Death	Death	Clinical improvement	Death	Death	Clinical improvement
Complaints		Fever, perilabial cyanosis, coma	Fever, convulsion	Fever, perilabial cyanosis, coma	Fever, changes of consciousness	Fever, cardiopulmonary arrest	Fever, convulsion	Fever, haematemesis, low response	Fever, convulsion	Fever, convulsion
Symptoms		Peak body temperature 40℃, coma	Peak body temperature 40℃, multiple GTC, non-projectile vomiting	Fever, dyspnea, coma	Peak body temperature 41–42℃, changes of consciousness	Peak body temperature 39.4℃, 2 GTCs	Peak body temperature 40.4℃, 3 GTCs, 1 vomiting	Fever, loss of consciousness, hypovolemic shock	Peak body temperature 40.3℃, non-projectile vomiting, 1 GTC	Peak body temperature 39.2℃, multiple GTC, projectile vomiting
Consciousness		Deep coma	Somnolence	Deep coma	Deep coma	Deep coma	Somnolence	Loss of consciousness	Somnolence	Somnolence
Blood test	WBC	21.01	8.25	13.37	11.18	14.95	8.08	8.06	9.3	6.71
	RBC	5.72	5.56	5.39	5.21	4.14	4.33	6.24	3.32	3.81
	PLT	26	152	35	52	179	110	298	52	112
	CRP	2.03	11.1	1.26	3.67	35.64	11.28		31.4	7.28
	PCT	5.19	38.37	4.75	3.34	2.26	14.22		41.53	25.43
	D-dimer	>56	37.96	6.45	>56	0.98	36.42		2.05	29
	IL-6	>5,000	97.7	>5,000	>5,000	35	11.7		1,968	57.5
	LDH	3,330.5	981.4	4,796.4	1,867.8	337.2	75		1,004.3	97
	ALT	55	104	845	225	68	250		191	1,873
	TBIL	3.7	3.6	8.8	12.2	8.4	7.2		5.5	2.8
CSF	CSF WBC						3		2	5
	CSF protein						4.5		1	3.36
Chest x-ray/Lung CT		Not done	Not done	Normal	Not done	Normal	Not done	Bilateral lung markings slightly thickened	Not done	Normal
Cranial CT		Cerebral edema	Not done	Cerebral edema	Cerebral edema	Not done	Multiple patchy low-density shadows in bilateral lateral ventricle, basal ganglia, thalamus, and brain stem	Not done	Cerebral edema	Reduced density in bilateral deep frontoparietal lobes, basal ganglia, and thalamus, complicated with bilateral thalamic edema
Cranial MRI							Multiple lesions in bilateral centrum semiovale, periventricular white matter of bilateral lateral ventricle, bilateral thalamus, callosum, cerebral peduncle, and dorsal brainstem, complicated with enhancement of leptomeninges			Multiple lesions in bilateral frontoparietal cortex, centrum semiovale, radial crown, basal ganglia area, lateral ventricle, splenium of corpus callosum, thalamus, pons, right cerebral peduncle and bilateral cerebellar hemisphere
Main treatment	MV	Yes	Yes	Yes		Yes		Yes	Yes	Yes
	Steroid	Yes	Yes			Yes	Yes		Yes	Yes
	IVIG		Yes						Yes	Yes
	TPE and/or CRRT								Yes	Yes
	Tocilizumab								Yes	Yes

## 4 Discussion

According to Hong Kong statistics, approximately 50 children needed to be treated at the pediatric intensive care unit (PICU) at the end of March 2022, accounting for 0.045% of the population under 19 years old. Among them, three deaths were reported from suspected encephalitis (accounting for 0.0027% of patients under 19 years old) (9). In addition, Taiwan Centers for Disease Control Complex reported that 6 out of 10 SARS-CoV-2 related deaths (60%) in children were attributed to fatal fulminant cerebral edema as of April 2022, with a total mortality rate of 0.0008% for people under 20 years old (4). Following a change in epidemic policy, a total of 36 children were admitted to the PICU of our hospital, 22 of whom presented with neurological symptoms. Therefore, the nine cases of critical neurological impairment included in this study exhibited a good representation.

It has been reported that patients infected with SARS-CoV-2 can experience various neurological complications, ranging from mild to severe, affecting both the central and peripheral nervous systems (10, 11). These complications can occur in both asymptomatic and severely affected COVID-19 patients due to different pathophysiological mechanisms, mainly characterized by encephalopathy, delirium and behavioral changes (12), while pulmonary symptoms are mild or absent. Herein, there were no significant changes in chest x-rays or lung CTs in any of the cases, indicating that the impact of the Omicron strain on the nervous system of children may be greater than its impact on the respiratory system.

Among the nine cases collected at our center, two cases were diagnosed with acute necrotizing encephalopathy (ANE) through early CSF testing and subsequent cranial MRI, while the remaining seven cases did not undergo CSF testing because of rapid disease progression. However, given the similarities in symptoms and signs across all cases, it is plausible that ANE was also the underlying cause of death in these cases. The pathogenesis of ANE remains unclear. The most popular hypothesis is that of cytokinemia, or a cytokine storm, which is a potentially lethal immune response consisting of a positive feedback loop between cytokines and leukocytes. Patients with ANE develop an exaggerated immune response to various viruses, similar to systemic inflammatory response syndrome (SIRS) (13).

Acute fulminant cerebral edema is a rare but severe complication of SARS-CoV-2 infection and a major pathological feature of acute necrotizing encephalopathy (ANE). Therefore, early identification of warning signs and close monitoring of clinical progression are crucial, given the potential for fatal outcomes (14). A previous study indicated that the SARS-CoV-2 Omicron variant might cause more febrile seizures in children (15). In our study, all patients exhibited seizures or sudden disorders of consciousness 24 h prior to the onset of symptoms of fulminant cerebral edema, accompanied by high or ultra-high fever. These findings suggest that the fever may be neurogenic due to hypothalamic damage or paroxysmal sympathetic hyperactivity (16). Therefore, children with SARS-CoV-2 infection should be closely monitored for acute fulminant cerebral edema if they exhibit early hyperpyrexia and neurological symptoms.

Consistent with our study, previous analyses have shown that elevated D-dimer and LDH levels are associated with encephalopathy and lung tissue injury, serving as potential early warning indicators for severe neurological changes (17). Our study also found that IL-6 levels significantly increased in patients with severe neurological impairment, consistent with the cytokine storm hypothesis (18), suggesting that using anti-inflammatory therapy to inhibit the cytokine storm may have a certain effect. The two children who survived were treated with gamma globulin and hormone pulse therapy in the early stage of the disease. One showed a rapid decrease in body temperature and an earlier recovery of consciousness after treatment, while the other child was additionally treated with continuous renal replacement therapy and tocilizumab. Both had central nervous system injury and different degrees of sequelae on MRI reexamination, but the former was milder, suggesting that the prognosis may be related to the symptom score at admission, and the role of continuous renal replacement therapy (CRRT) in eliminating inflammatory factors may delay disease progression. In addition, there is a lack of comprehensive data analysis worldwide supporting the definite efficacy of blood purification and tocilizumab (19). Cheng et al. (20) previously reported a successful case of using therapeutic plasma exchange (TPE) combined with continuous renal replacement therapy (CRRT) to treat a critically ill child with COVID-19. A previous research report indicated satisfactory recovery in three pediatric cases of ANE after early intravenous administration of methylprednisolone and tocilizumab monoclonal antibody (21). A review of case reports of patients who developed COVID-19 pneumonia and subsequently progressed to ANE revealed that these patients did not receive tocilizumab treatment. Therefore, similar treatment options were adopted in the management of the cases involved in this study (22). In current research, two pediatric patients also underwent the combined therapy. Although one child died of bacterial infection, IL-6 and other indicators decreased rapidly after early blood purification treatment, indicating that blood purification has a certain positive effect in managing cytokine storms (20). Teoh et al. analyzed the clinical features and outcomes of 16 children and young adults with severe acute COVID-19 treated with tocilizumab and found that delayed administration after admission to PICU may be an important predictor of poor prognosis (23). One of our patients died of a severe bacterial infection (Escherichia coli) after blood purification and tocilizumab treatment. A possible reason could be due to secondary infection caused by the immunosuppressive effect of tocilizumab; however, further studies are warranted to determine the side effects of tocilizumab. A case report from Singapore described an 11-year-old boy with acute SARS-CoV-2 infection who presented with ophthalmoplegia, ataxia, and aphasia, highlighting the diverse and potentially severe neurological manifestations of COVID-19 in children. The neuroimaging results confirmed significant edema and signal changes in the bilateral thalamus, brain stem, and cerebellar hemispheres, consistent with childhood ANE. The combination of early steroid therapy, intravenous immunoglobulin (IVIG), and targeted IL-6 blocking therapy clearly improved neurological function (24).

Immune responses play a crucial role in many neurological complications and post-infectious manifestations of COVID-19. Therefore, when children, especially infants, present with symptoms such as seizures, altered mental status, or hyperpyrexia, the possibility of acute encephalopathy should be considered, and early initiation of relevant immunotherapy may be beneficial (20). However, the use of steroids in the treatment of lung injury in COVID-19 has been controversial. Baillie et al. previously suggested that steroids have little impact on mortality in COVID-19 patients with septic shock and are generally not useful in shock management (25). Cao et al. concluded that overwhelming inflammation and cytokine-related lung injury among critically ill patients might lead to rapid progressive pneumonia and recommended the cautious use of glucocorticoids (26). For the severe neurological impairment caused by SARS-CoV-2, no study with a large sample size has been conducted on the efficacy of corticosteroids. Still, we believe that cytokine storm remains the main pathological mechanism of fulminant cerebral edema. According previously reported studies (27–30), the potential mechanisms we believe to be involved primarily encompass four aspects: (1) Infection and post-infectious hyperinflammatory response or autoimmune damage: COVID-19 infection leads to immune dysregulation, characterized by high levels of pro-inflammatory and anti-inflammatory cytokines, chemokines, and complement activation. It may also induce autoimmune abnormalities through molecular mimicry, which could be a primary mechanism for severe neurological involvement; (2) Direct viral invasion: This could occur through neurotropic spread or hematogenous dissemination, and can disrupt the integrity of the blood-brain barrier and induce excessive barrier permeability; (3) Vascular endothelial injury and hypercoagulable state: SARS-CoV-2 inhibits endothelial cell mitochondrial function and nitric oxide synthase activity by binding to angiotensin-converting enzyme 2 (ACE2), damaging vascular endothelial cells and affecting cerebrovascular and cardiac functions. Meanwhile, complex mechanisms such as endothelial disease, hypoxia induced by pneumonia, neutrophil extracellular trap release, and massive inflammatory responses can also lead to a hypercoagulable state; (4) Nervous system damage caused by pulmonary or systemic dysfunction: examples include hypoxemia and acute encephalopathy resulting from multi-organ dysfunction. Furthermore, glucocorticoids have a certain effect in inhibiting the inflammatory response, but secondary infection should be carefully monitored.

This study has some limitations. For instance, only nine cases were included in this research. Given the rarity of the cases, the issue of insufficient sample size is expected to be further supplemented by more clinical data reports. However, in the context of the ongoing COVID-19 pandemic, considering the high disease progression and malignancy of these cases, although it is not possible to fully complete all clinical data, this study still holds great reference value for subsequent clinical practitioners. Therefore, the focus of this article is to elaborate on the clinical symptoms and treatment regimens of these nine pediatric patients in the form of a case report, hoping to provide a reference for subsequent clinical practitioners.

The COVID-19 pandemic may have subsided, but the lasting neurological damage caused by SARS-CoV-2 in children has provided valuable insights and experiences in treating acute fulminant cerebral edema. As a novel virus, many mechanisms underlying the related complications of COVID-19 remain unclear. In the future, multicenter studies can be initiated to conduct large-scale controlled studies on the pathogenesis and treatment of acute encephalopathy caused by COVID-19 and other viruses, with the aim of validating the therapeutic effects and prognosis of immunotherapy on acute encephalopathy induced by viruses through a more substantial number of cases.

## Data Availability

The original contributions presented in the study are included in the article/Supplementary Material, further inquiries can be directed to the corresponding author.
